# Managing Scoliosis in a Young Child with Rett Syndrome: A Case Study

**DOI:** 10.1100/tsw.2005.33

**Published:** 2005-03-29

**Authors:** Meir Lotan, Joav Merrick, Eli Carmeli

**Affiliations:** ^1^ Department of Physical Therapy, Haifa University, Haifa and Therapeutic Department, Zvi Quittman Residential Center, Millie Shime Campus, Elwyn, Jerusalem, Israel; ^2^ National Institute of Child Health and Human Development, Faculty of Health Sciences, Ben Gurion University of the Negev, Beer-Sheva, Office of the Medical Director, Division for Mental Retardation, Ministry of Social Affairs, Jerusalem, Israel; ^3^ Department of Physiotherapy, Sackler Faculty of Medicine, Stanley Steyer School of Health Professions, Tel Aviv University, Ramat Aviv, Israel

**Keywords:** Rett syndrome, scoliosis, conservative management, regression of scoliosis, Israel

## Abstract

Rett syndrome is a genetic disorder primarily affecting females. One of its most disabling features is the severe and rapid progression of scoliosis. So far, only surgical intervention has succeeded in reversing the development of scoliosis in Rett syndrome.The present study describes a new management approach implemented with a girl with Rett syndrome. The core of the management regime was intensive: asymmetrical activation of trunk muscles through equilibrium reactions. The X-rays accompanying the article (evaluated by four experienced orthopedic surgeons blinded to the intervention process) suggested that the intervention was successful in reversing the progress of the scoliosis for the above-mentioned child. Discontinuation of treatment led to severe and rapid deterioration of the spinal curve.Due to the fact that this was a case study, generalization is limited, but we suggest further investigation and studies with this method.

## INTRODUCTION

Rett syndrome is a genetic disorder that primarily affects females. The disorder causes a neurological and developmental arrest that manifests itself in a variety of disabilities such as loss of functional hand use, loss of acquired speech, apraxia, ataxia, autonomic system dysfunction, epilepsy, failure to thrive, and muscle tone irregularities[1]. One of the debilitating features of Rett syndrome is a severe and rapidly progressing neuromuscular scoliosis. The term “neuromuscular scoliosis” describes curves that result from neurological disorders[2]. Neuromuscular scoliosis appears at an early age and develops rapidly, and unlike idiopathic scoliosis, cessation of physical growth does not halt the progression of the scoliosis[1]. “The cause for scoliosis in the neurological patient” is enigmatic and “could be: superimposed by asymmetrical spinal muscle contractions… a result of sensory defect … muscle contracture /involvement…"[3, pg. 445] or weakness[4]. In Rett syndrome, scoliosis is apparent in up to 80–85% of adult females with Rett syndrome[1,6]. Often, the neuromuscular curvature is characterized by a long "C-type" curve, but as compensatory curves become structural, the pattern may change to an "S-type" curve[5], as is the case in 88% of this population[1].

The average annual progression rate of scoliosis in Rett syndrome is 14º[1,6] and there are no known reports of spontaneous curve regression. Better prognosis for scoliosis in Rett syndrome is observed when kyphosis, normal muscle tone, and tendon reflexes are apparent[1] and the ability to climb up and down stairs independently or with assistance is obtainable[6]. The prognosis for scoliosis in Rett syndrome is worse when it appears before the age of 5, when severe hypotonia exists, and when there is an inability to walk or the loss of the ability to walk at an early age[1].

"Non-surgical evaluation and management of neuromuscular spinal curvature include orthopedic clinical observation, radiographic examination"[5, pg.250], electrical paraspinal functional neuromuscular stimulation[7], and orthotic management[3,4,5,8,9]. Although no research studies have shown that custom seating is effective in reducing or preventing progression of curves, and although orthosis eliminates the normal flexibility of the spine… it is an important part in clinical treatment [3,5]. Treatment strategies suggested to date for treating scoliosis in Rett syndrome include the following:

- Intensive physical and hydrotherapy treatments — Reported to yield the maximal benefits according to the results of a questionnaire[6].
- Aggressive treatment starting as soon as the first spinal asymmetry is noticed — Suggested by McClure et al.[10], who found a close correlation between the primary asymmetry of the back and the later developing scoliosis.
- Intensive walking or standing — if the girl is non-mobile, should be implemented for at least half an hour a day[11].
- "Overcorrection" treatment — Applied in order to readjust the skewed midline perception of the females manifesting scoliosis[12].
- Corsets and temporary casting — When used, were mostly reported as unsuccessful in changing the progression of scoliosis in females with Rett syndrome[6,13].
- Surgical intervention — The only method reported to date that successfully halts and reversed the progression of the scoliotic curve[6].

## CASE REPORT

TR, an 8-year-old girl with Rett syndrome, was enrolled at a special education/habilitation center where she received traditional intervention, which included bi-weekly physical therapy, bi-weekly hydrotherapy, and adjusted seating. However, when the outcome of the intervention was assessed, the results showed no change in the advancement of the scoliotic curve. These findings encouraged the therapist to attempt a novel intervention. This new approach was tested in full with a different child with Rett syndrome, called RC.

At the end of June 1998, when RC was 5 years old, she began showing slight signs of spinal asymmetry. RC was also showing all the predictors of a bad prognosis: she was young, showed severe hypotonia, and had never walked[1]. By August of the same year, she was showing a 29º Cobb right C scoliosis in an X-ray taken in supine position (see Fig. 1). A follow-up X-ray taken about 1 year later in a suspended position showed a 22º Cobb right C scoliosis (see Fig. 2). The difference in postures in both X-ray exposures prevented comparison between the two.

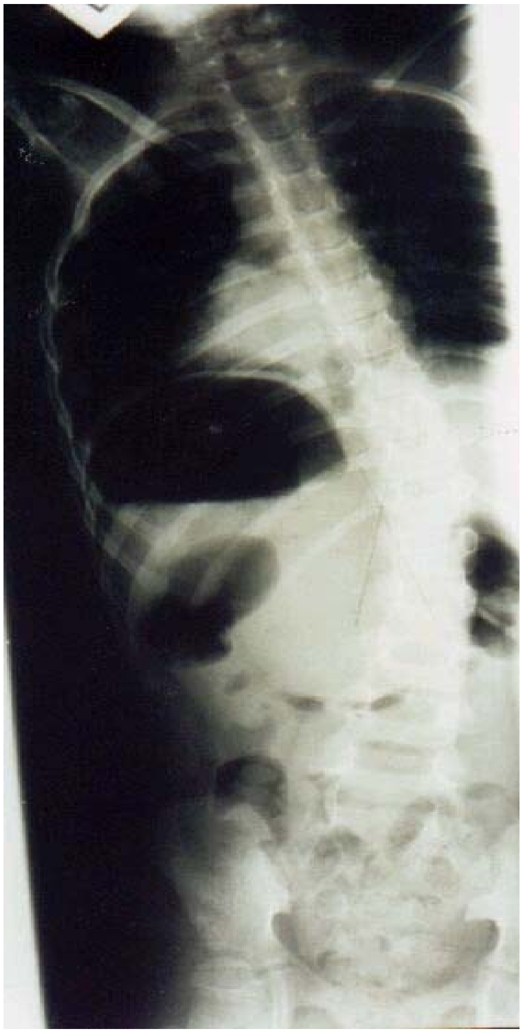

**FIGURE 1**. A 29º Cobb right C-shaped scoliosis taken in a supine position on August 2, 1998.

The rapidly advancing scoliosis made it necessary to take immediate intensive steps to develop a new management plan. The fact that 88% of females with Rett syndrome show an S-shaped scoliosis led the therapist to believe in their ability to correct the spine's tendencies (the belief was that the scoliosis started as C-shaped and the child's efforts to correct her posture will eventually cause her to end up with an S-shaped scoliosis). Physical examination showed that RC's equilibrium reactions (equilibrium reactions are those highly integrated complex automatic responses to changes in posture and movement aimed towards restoring balance and preventing falls with test administered according to Uyanik et al.[14]) were present and active in a seated position, but not quick enough to be considered efficient in fall prevention. The new intervention was based on the following principles (some are based on existing evidence, but most are new concepts):

- Follow up visits at orthopedic surgeon twice a year[5,10]
- Commencing treatment as soon as asymmetry of the spine is detected[10]
- Intensive implementation throughout sleeping and waking hours
- Opposing (to the natural scoliosis curve) asymmetry postures
- Walking and/or standing at least 2 h a day[11]
- Maintaining spinal mobility through passive manual manipulation
- Parental and staff guidance

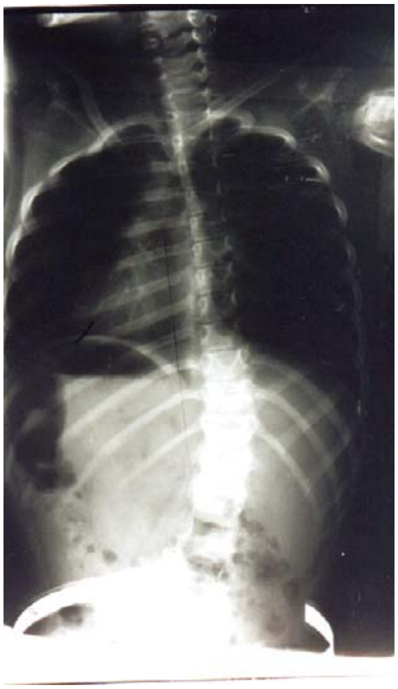

**FIGURE 2**. A follow-up X-ray, taken on July 18, 1999, in a suspended position showing 22º Cobb

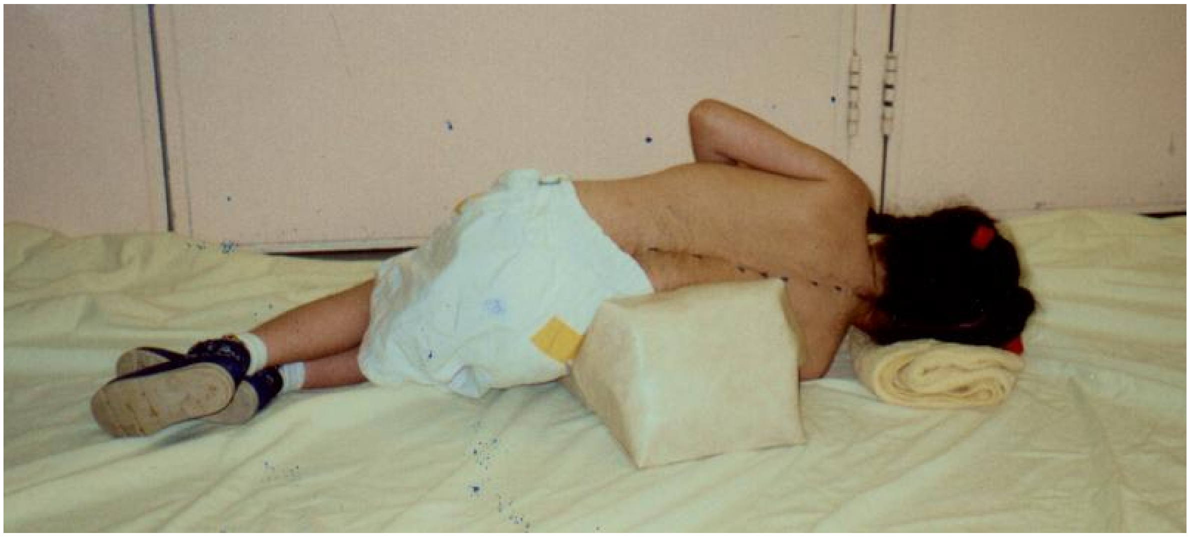

**Photograph 1**. A special cushion.

Implementing the program made it necessary to develop new and unique equipment such as a U-shaped pillow (to be used during sleep/nap times, see Photo 1), a special seat (see Photo 2), an adapted chair (see Photo 3), and an adapted standing frame (see Photo 4), all tilted forward and to the left, causing the child to use balance reactions by working asymmetrically — harder with the extensors and side flexors of the right side of her body — against the natural pull of the scoliosis. As mentioned earlier, the program was very intense and the child's weekly schedule included asymmetrical positioning throughout the day (9:00–15:00) except for meal times (see Table 1). It should be emphasized that the intensity of the program did not impede the child's educational curriculum.

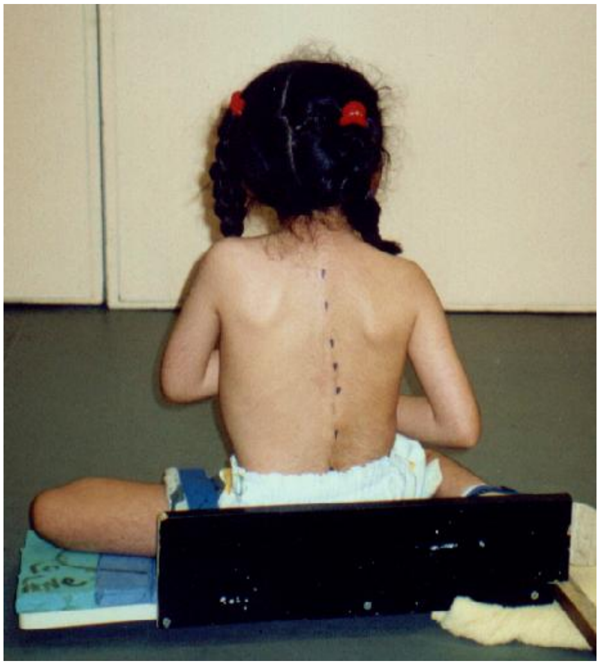

**Photograph 2**. A special seat.

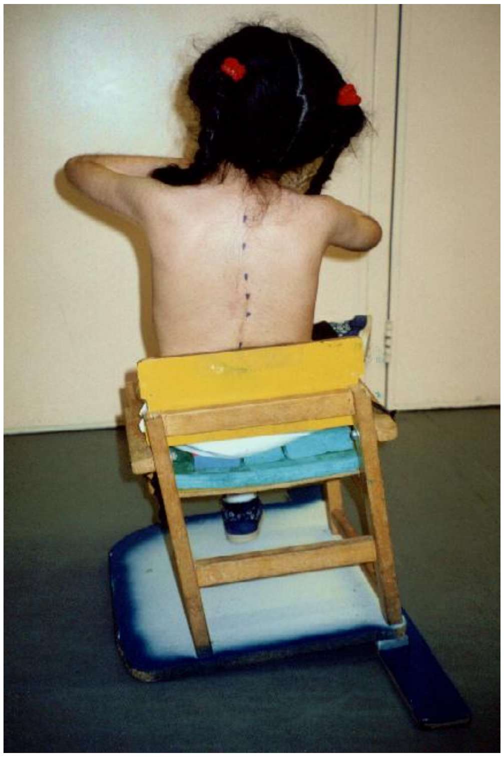

**Photograph 3**. A special chair.

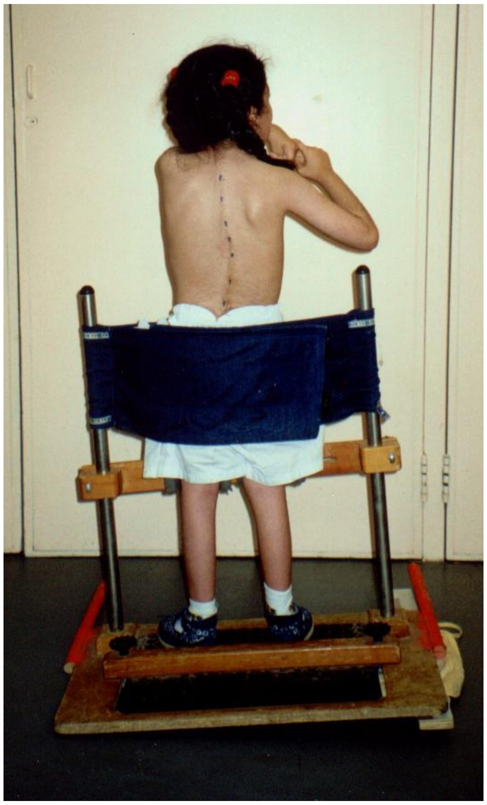

**Photograph 4**. A special standing frame.

**TABLE 1**
**Child's weekly schedule**

<table>
<tr><th>Days<br>Hours</th><th>Sunday</th><th>Monday</th><th>Tuesday</th><th>Wednesday</th><th>Thursday</th><th>Friday</th></tr>
<tr><td>0800-08:30</td><td></td><td></td><td></td><td></td><td></td><td></td></tr>
<tr><td>08:30-09:00</td><td>Hydrotherapy</td><td>Physical therapy</td><td></td><td>Physical therapy</td><td>Hydrotherapy</td><td></td></tr>
<tr><td>09:00-09:30</td><td colspan="6">SPECIAL CHAIR</td></tr>
<tr><td>09:30-10:00</td><td colspan="6">STANDING FRAME</td></tr>
<tr><td>10:00-10:30</td><td></td><td></td><td></td><td></td><td></td><td></td></tr>
<tr><td>10:30-11:00</td><td colspan="6">WALKING</td></tr>
<tr><td>11:00-11:30</td><td colspan="6">SPECIAL SEAT</td></tr>
<tr><td>11:30-12:00</td><td colspan="5">SPECIAL CHAIR</td><td></td></tr>
<tr><td>12:00-12:30</td><td></td><td></td><td></td><td></td><td></td><td></td></tr>
<tr><td>12:30-13:00</td><td></td><td></td><td></td><td></td><td></td><td></td></tr>
<tr><td>13:00-13:30</td><td colspan="5">SIDE LYING</td><td></td></tr>
<tr><td>13:30-14:00</td><td colspan="5">SPECIAL CHAIR</td><td></td></tr>
<tr><td>14:00-14:30</td><td colspan="5">STANDING FRAME</td><td></td></tr>
<tr><td>14:30-15:00</td><td></td><td></td><td></td><td></td><td></td><td></td></tr>
</table>

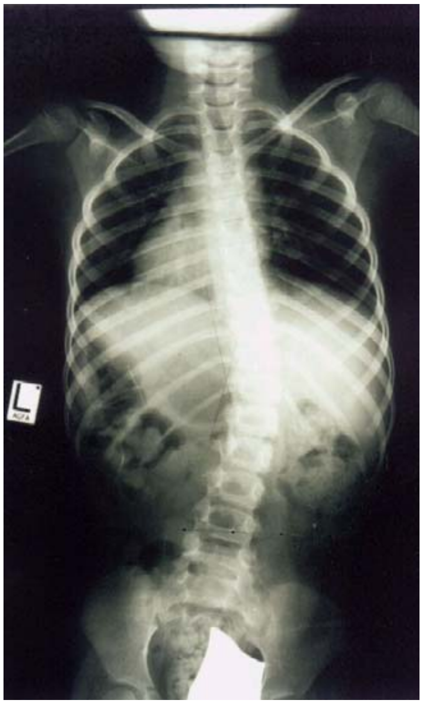

**FIGURE 3**. X-ray taken on February 16, 2000, in supine position showing 20º Cobb.

After a year and a half of intervention, two X-rays were performed (Figs. 3 and 4). Since the X-rays were taken at different dates and in different positions, an explanatory summarizing graph was added (Graph 1). The X-rays sets where evaluated by four experienced orthopaedic surgeons, who did not know the child and were blinded to the intervention procedure and the X-ray dates (see Table 2). Each evaluator was individually introduced to the four X-ray photos and asked to measure the Cobb angle[15]. The findings of the evaluators support the fact that the treatment produced positive results in reversing the course of the scoliosis.

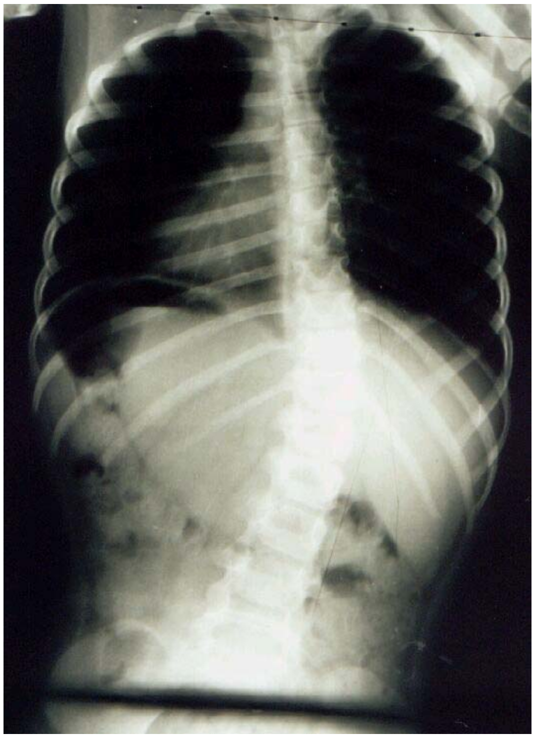

**FIGURE 4.** X-ray taken on February 16, 2000, in a suspended position showing 20º Cobb.

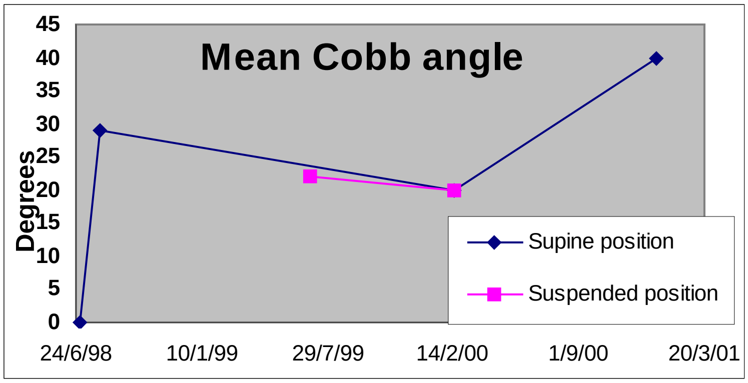

**Graph 1.** Development of scoliosis according to consecutive X-rays.

Due to a change in the child's educational setting, the above-mentioned approach was discontinued. One year after terminating the management approach described in the present article, the child was measured at a Cobb angle of 40º (X-ray unavailable) and a corset was ordered.

## CONCLUSION

The majority of conventional interventions that manage scoliosis in Rett syndrome have so far showed the ability to slow the curve progression, but there are no existing reports on an intervention that was able to reduce the Cobb angle of a scoliotic curve in this population. The failure of the conventional management

**TABLE 2**
**Average X-Ray Measurement**

| Observer # | Individual Measurement | Average Measurement |
|---|---|---|
| X-ray taken on 8/2/1998, supine position | | |
| 1 | 28 | |
| 2 | 26 | 29 |
| 3 | 30 | |
| 4 | 31 | |
| X-ray taken on 7/1/1999, suspended position | | |
| 1 | 22 | |
| 2 | 21 | 22 |
| 3 | 23 | |
| 4 | 22 | |
| X-ray taken on 2/16/1999, supine position | | |
| 1 | 22 | |
| 2 | 20 | 20 |
| 3 | 18 | |
| 4 | 20 | |
| X-ray taken on 2/16/1999, suspended position | | |
| 1 | 20 | |
| 2 | 21 | |
| 3 | 19 | 20 |
| 4 | 20 | |

approach led to a new intervention regime. The treatment described in this study was an intense regime that necessitated adapted equipment, but mostly required a vigorous and cooperative caregiving team. The results seemed to indicate that this intervention might be effective in treating scoliosis in Rett syndrome. As this was a case study, generalization is limited. It should be noted that the results were achieved in one girl, who had no self-propulsion abilities. This means that the constant external positioning by a team of caregivers did not jeopardize any of her chances to move independently. Unfortunately, since her treatment terminated, a rapid spine deterioration was detected in the course of one year. We believe that treatment continuation until the final stages of skeletal development might have minimized the expected deterioration of her spinal curves. Further investigation on this topic is warranted.

## BIOSKETCHES

**Meir Lotan, BPT, MScPT,** is a physiotherapist working at the Zvi Quittman Residential Center, The Millie Shime Campus, Elwyn Jerusalem with special interest in physiotherapy aspects on intellectual disability, Snoezelen, and physical activity for children and adults with intellectual disability. He lectures on assistive technology at the Department of Physical Therapy, Haifa University and Ben Gurion Univeristy. E-mail: ml_pt_rs@netvision.net.il

**Eli Carmeli, BPT, PhD**, is currently a Senior Lecturer of Gerontology and Anatomy and the chairman of the Department of Physical Therapy Department, Stanley Steyer School of Health Professions, Sackler Faculty of Medicne, Tel Aviv University. He is also the Editor-in-Chief of the Israeli Journal of the Physiotherapy Society. Eli received his BPT degree from Sackler Faculty of Medicine, Tel Aviv University and his PhD degree from the Rappaport Faculty of Medicine at the Technion in Haifa. Postdoctoral research was conducted at the University of Florida in Gainesville. His research interests are investigating the aging process both on the cellular and clinical level. E-mail: elie@post.tau.ac.il Website: http://www2.tau.ac.il/Person/medicine/HealthSchool/researcher.asp?id=agfhfiffl

**Joav Merrick, MD, DMSc,** is professor of child health and human development affiliated with the Zusman Child Development Center, Division of Pediatrics and Community Health at the Ben Gurion University, Beer-Sheva, Israel; the medical director of the Division for Mental Retardation, Ministry of Social Affairs, Jerusalem; founder and director of the National Institute of Child Health and Human Development. He has numerous publications in the fields of child health and human development, rehabilitation, intellectual disability, disability, health, welfare, abuse, advocacy, quality of life, and prevention. Dr. Merrick received the Peter Sabroe Child Award for outstanding work on behalf of Danish Children in 1985 and the International LEGO-Prize ("The Children's Nobel Prize") for an extraordinary contribution towards improvement in child welfare and well being in 1987. E-mail: jmerrick@internet-zahav.net. Website: www.nichd-israel.com
